# Simultaneous ectopic adrenocorticotropic hormone syndrome and adrenal metastasis of a medullary thyroid carcinoma causing paraneoplastic Cushing's syndrome

**DOI:** 10.1186/1477-7800-4-15

**Published:** 2007-07-02

**Authors:** Michael Sand, Samuel Uecker, Falk G Bechara, Marcos Gelos, Daniel Sand, Till H Wiese, Benno Mann

**Affiliations:** 1 Department of General and Visceral Surgery, Augusta Krankenanstalt, Academic Teaching Hospital, Ruhr-University Bochum, Germany; 2 Department of Dermatology and Allergology, Ruhr-University Bochum, Germany; 3 Department of Physiological Science, University of California Los Angeles (UCLA), USA; 4 Department of Radiology, Augusta Krankenanstalt, Academic Teaching Hospital of the Ruhr-University Bochum, Germany

## Abstract

Medullary thyroid carcinomas (MTC) constitute about 5 to 7 % of thyroid neoplasms. They originate from parafollicular C-cells which can secrete adrenocorticotropic hormone (ACTH) and/or corticotropin-releasing factor (CRF) in abnormally high concentrations, potentially causing paraneoplastic Cushing's Syndrome (CS).

We report on a 42-year-old male patient with a ten year history of metastatic medullary thyroid carcinoma suffering from paraneoplastic Cushing's Syndrome caused by ectopic hypersecretion of ACTH and a simultaneous Cortisol producing adrenal metastasis.

## Background

Medullary thyroid carcinoma (MTC) is relatively rare and represents 3% to 10% of all thyroid carcinomas [1]. It is the third most common of all thyroid cancers. This tumor entity derives from parafollicular C-cells of the thyroid which are of neural crest origin and secrete calcitonin, a polypeptide hormone which is an excellent tumor marker for diagnosis and post-surgical follow-up, as it represents the biochemical activity of MTC [2-4].

MTC can occur both in genetic or sporadic form. The genetic form can either be isolated or as part of multiple endocrine neoplasia (MEN) type 2A- (Sipple-) or 2B- (Gorlin-) syndrome. Independent from its form, humorally mediated symptoms may rarely be associated with MTC. The C-cells potentially produce peptides such as adrenocorticotropic hormone (ACTH) and/or corticotropin-releasing factor (CRF) in abnormally high concentrations which may lead to paraneoplastic Cushing's Syndrome (CS). Ectopic hypersecretion of ACTH leading to paraneoplastic Cushing's Syndrome is a rare occurrence in patients with MTC. Only 50 cases have been reported before now [5-9].

Another possible cause for Cushing's Syndrome is an adrenal gland tumor primary or as a metastasis [10]. The combination of both in a single patient has not been reported to date.

## Case presentation

We report on a 42-year-old male patient with a 10-year history of MTC. He suffered from abdominal, retroperitoneal, osseous, pulmonary and mediastinal metastases. The patient underwent multiple tumor and metastases resections within the past 10 years. He received a modified radical neck dissection and a clearance of the upper mediastinum due to progressive tracheal compression. His family history was unremarkable regarding any endocrinological or thyroid disease. Specifically, a familial form of MTC was excluded. He suffered from strong persisting diarrhea which could only be controlled with opium medication (tinctura opii 60 drops/d).

Three weeks before referral to our unit he developed multiple episodes of strong muscular weakness which was temporarily so extreme that it led to paralysis. He developed diabetes and laboratory values showed very low potassium levels, necessitating continuous intravenous potassium replacement. Additionally he had exorbitantly high levels of calcitonin > 2000 pg/ml and low levels of calcium (2.34 mmol/l). Laboratory findings revealed hypercortisolism, with cortisol values > 630 ng/ml (normal range; 23–119 ng/ml). The ACTH values were elevated > 470 ng/l (normal range; 6–30 ng/l). A CT-scan of the head showed a normal pituitary gland, excluding a pituitary tumor.

A dexamethasone short- and long-term suppression test showed no decrease of cortisol or ACTH. The rare diagnosis of a secondary Cushing's Syndrome with ectopic ACTH-production due to a metastasized MTC was established.

An adrenostatical treatment with the maximum dose of 1200 mg ketoconazole per day was initiated. After three weeks the metabolic situation of the patient did not change. The high values of free cortisol in the urine persisted (4,000 to 10,000 μg/d). He further developed persisting low potassium levels and unstable blood glucose values which were treated with up to 500 mmol potassium/24 h and regular insulin up to 250 IE/24 h.

**Figure 1:**
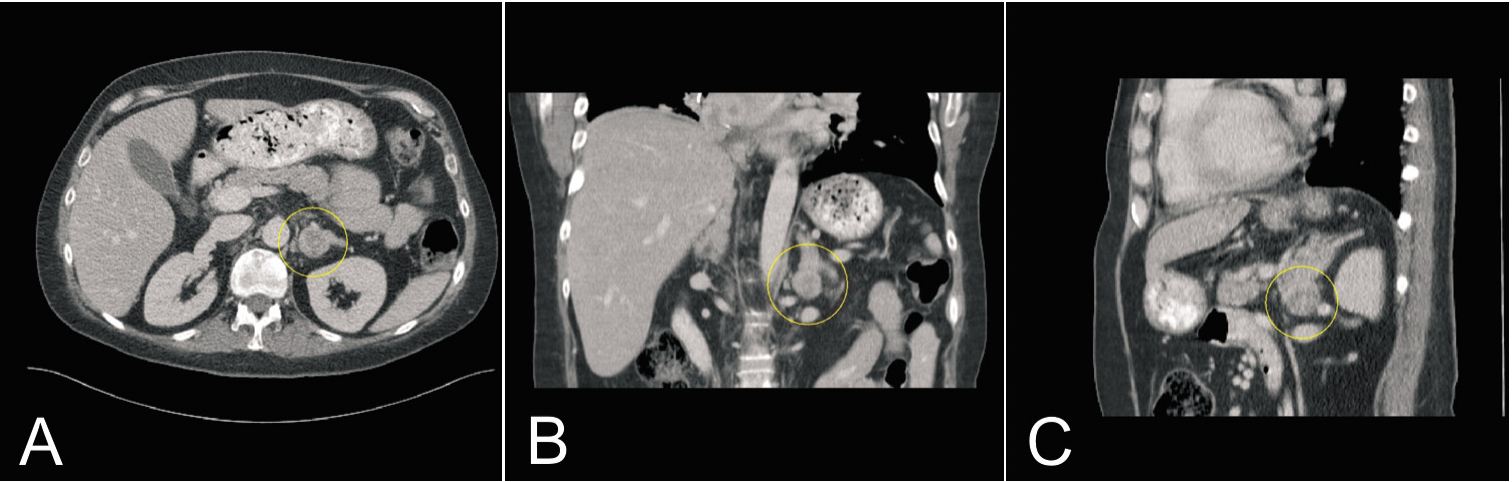
Abdominal CT scan demonstrating the horizontal (a), coronal (b) and sagittal (c) section of a tumor in the left adrenal gland (circle).

In order to further investigate the cause of this disordered metabolism, a CT-scan of the abdomen was performed. Besides abdominal, retroperitoneal, osseous, pulmonary and mediastinal metastases a tumor in the left adrenal gland of 2 cm in diameter was described (Fig. 1). Both adrenal glands were hypertrophic with a diameter of 5 × 2 × 3 cm. In order to determine the cortisol and ACTH levels in various veins of the body selective venous catheterisation was used to anatomically localize the ectopic ACTH-production. All regions of the body revealed consistently high values of ACTH (180 to 220 ng/l) and cortisol (580 to 630 ng/ml).

To eliminate the production of excess cortisol we performed a bilateral endoscopic retroperitoneal adrenalectomy. The bilateral hyperthrophy of the adrenal glands was verified intraoperatively and the left adrenal gland showed a tumor-like nodule. Both adrenal glands were removed. Histopathologic findings showed a metastasis in the left adrenal gland originating from the MTC. Immediately after the operation a substitution of 200 mg hydrocortisol per day was started. Unfortunately the need for potassium and insulin did not change as compared to the levels substituted before the operation. After ten days the hydrocortisol substitution was stopped for 8 h. As a consequence the cortisol levels normalized. Therefore we interpreted the high demand for potassium and insulin as a result of the cortisol substitution. The level of hydrocortisol substitution was reduced to 100 mg/d (intravenous) and 4 × 20 mg/d for two days and then stopped again. Postoperatively the patient developed a chest infection and subsequently entered respiratory failure. He was transferred to the intensive care unit, but despite aggressive treatment, died a few days later.

## Discussion

Cushing's Syndrome is a hormonal disorder caused by prolonged exposure of the body's tissues to high levels of cortisol. The most common cause of hypercortisolism (80%) is a pituitary adenoma (Cushing's disease), which was excluded in our patient [11]. Another possible cause is ectopic ACTH- and/or CRH-Syndrome caused by benign or malignant tumors originating outside the pituitary gland (10–15%). This condition is described as ectopic, paraneoplastic ACTH and/or CRH production and is mostly induced by lung tumors (50%). The most common forms of ACTH-producing tumors are oat cell or small cell lung cancer which accounts for about 25% of all lung cancer cases, and carcinoid tumors. In rare cases less common types of tumors which are described causing ectopic ACTH production are: pancreatic islet cell tumors, thymomas, very rarely abdominal carcinoids, liver-, prostate-, breast- or colon and medullary thyroid carcinomas. Paraneoplastic, ectopic hypersecretion of ACTH is a very rare occurrence in MTC, with only 50 cases reported.

MEN I can cause Cushing's Syndrome due to pituitary, ectopic or adrenal tumors, and has to be diagnosed by PCR genotype analysis when suspected. Other possible causes of CS are adrenal gland tumors which often lead to rapid development of clinical symptoms. Most of these cases involve adrenal adenomas. Adrenal cancers, primary or as metastasis are one of the least common causes of Cushing's Syndrome [12].

Cushing's Syndrome and, much more frequently, patients receiving exogenous glucocorticoid therapy (after adrenalectomy for example), are especially at risk of infections. The same is true for patients with ectopic ACTH-production which potentially leads to extensive hypercortisolism as in our patient. This vulnerability is attributed to the complex dysregulation of immunity caused by glucocorticoids [13]. The cytotoxic T-lymphocyte type 1: type 2 ratio is impaired which makes patients prone to serious opportunistic infections [14].

In the author's point of view it is important to be aware of early clinical and laboratory signs of ectopic Cushing's Syndrome and further initiate laboratory and radiologic examinations determining the causes of excess levels of cortisol.

The diagnosis of paraneoplastic CS is based on hypercortisolism with normal or high levels of plasma ACTH and elevated lipotropic pituitary hormone (LPH), which is not suppressed by low-dose dexamethason suppression, and the absence of pituitary adenoma as seen in pituitary MRI. Both were the case in our patient. Uncommonly, the diagnosis is confirmed by immunohistochemical techniques or by proopiomelanocortin mRNA in situ hybridization of MTC tissue.

Therapy is either conservative or surgical. Somatostatin analogues, metyrapone, aminoglutethimide, etomidate and ketoconazol are among the medications described in the literature [15,16]. The indication for bilateral adrenalectomy, however, should be discussed early in the disease. As in our patient a conservative form of therapy was ineffective and valuable time was lost before we made the diagnosis of an adrenal gland metastasis.

Surgical treatment of the patient's cancer may lead to cure. However, in patients with advanced disease and metastases, as was the case described, the early diagnosis of CS would be of strong value as specific treatment of hypercortisolism including bilateral adrenalectomy, can be initiated early, before the impact on the patient's immunity leads to serious complications.

Persistent diarrhea has been described as a symptom of MTC [17]. This phenomenon is possibly linked to the involvement of calcitonin gene-related peptide (CGRP) which has been shown to induce colonic fluid secretion in the rat colon with an increased secretion of sodium, potassium, and chloride [18,19]. The extent to which the latter observation can be valid for humans has yet to be investigated.

## Conclusion

In order to decrease the likeliness of infection and improve survival it would be beneficial to screen patients with MTC for early signs of a developing CS. Although it is extremely rare it has to be kept in mind that in cases when conservative forms of therapy show no effect, a second cause for the abnormally high cortisol values, such as an adrenal gland tumor is possible and should be excluded.

However, in the context of our patients' disseminated metastases, it can not be finally assessed if his adrenal metastasis was an incidental finding or conclusively responsible for the CS. Bearing in mind that our patient developed his severe symptoms over a short period of time after 10 years of disease makes the adrenal gland metastases the most likely cause for CS. An earlier recognition and treatment of CS should help in prevention of opportunistic infections by potentially decreasing the fatal impact of hypercortisolism on the patient's immunity.

## Competing interests

All authors hereby disclose any commercial associations which might pose or create a conflict of interest with information presented in this manuscript. All authors declare that they have no competing interests.

## Authors' contributions

MS: documented and prepared the draft

SU: documented and prepared the draft

MG: Surgeon who performed the operations and helped in preparing the manuscript

FGB: Literature search and edited part of the manuscript

THB: Edited part of the manuscript and interpreted radiologic images.

DS: Literature search, revision of bibliography and edited most of the manuscript

BM: Surgeon who performed the operations and edited part of the manuscript and helped in preparing the draft

All authors read and approved the final manuscript.
